# Alkamide Affinin Regulates Adipogenesis Parameters in Insulin-Sensitive and Insulin-Resistant 3T3-L1 Adipocytes

**DOI:** 10.3390/molecules31213793

**Published:** 2026-10-22

**Authors:** Anaguiven Avalos-Soriano, María José Soto-López, Jesús Eduardo Castro-Ruíz, Ulisses Moreno-Celis, Teresa García-Gasca, María Soñadora Niño-Castañeda, Nadia Denys Betancourt-Martínez, Javier Morán-Martínez

**Affiliations:** 1 Secretary of Science, Humanities, Technology, and Innovation (SECIHTI)-Department of Cell Biology and Ultrastructure, Laguna Unit Faculty of Medicine, Autonomous University of Coahuila (FM-UL, UAdeC), Av. Morelos No. 900 Ote., Torreón 27000, Coahuila, Mexico; 2 Genotoxicology Laboratory, Autonomous University of the West (UAdeO), Los Mochis Regional Unit, Los Mochis 81200, Sinaloa, Mexico; 3 School of Dentistry, Faculty of Medicine, Autonomous University of Querétaro, Querétaro 76176, Querétaro, Mexico; 4 Faculty of Natural Sciences, Autonomous University of Querétaro, Av. de las Ciencias S/N, Juriquilla 76230, Querétaro, Mexico; 5 Department of Cell Biology and Ultrastructure, Laguna Unit Faculty of Medicine, Autonomous University of Coahuila (FM-UL, UAdeC), Av. Morelos No. 900 Ote., Torreón 27000, Coahuila, Mexico

**Keywords:** adipogenesis, alkamide affinin, 3T3-L1 preadipocytes, type 2 diabetes, rosiglitazone, 2-NBDG uptake, insulin sensitivity

## Abstract

This study investigated the effect of a crude extract of the alkamide affinin on various parameters related to adipogenesis and type 2 diabetes, using an in vitro cell culture system. The maximum non-toxic concentration of the extract was determined in 3T3-L1 cells, and its effect was evaluated in both normal cells and insulin-resistant adipocytes. In normal cells, the extract significantly reduced adipogenesis. In insulin-resistant adipocytes, no increase in triglyceride synthesis was observed compared to the resistant control. The extract stimulated the uptake of the glucose analog 2-NBDG in insulin-resistant adipocytes. Glucose uptake was higher than that observed in the resistant control group and the group treated with rosiglitazone, an insulin-sensitizing drug. The results indicate that affinin improves insulin response by promoting glucose uptake in insulin-resistant adipocytes. Furthermore, it did not show significant pro-adipogenic effects; conversely, it demonstrated insulin-sensitizing activity. Overall, the crude affinin extract reduced adipogenesis and stimulated glucose uptake without causing appreciable pro-adipogenic effects.

## 1. Introduction

**Figure 1:**
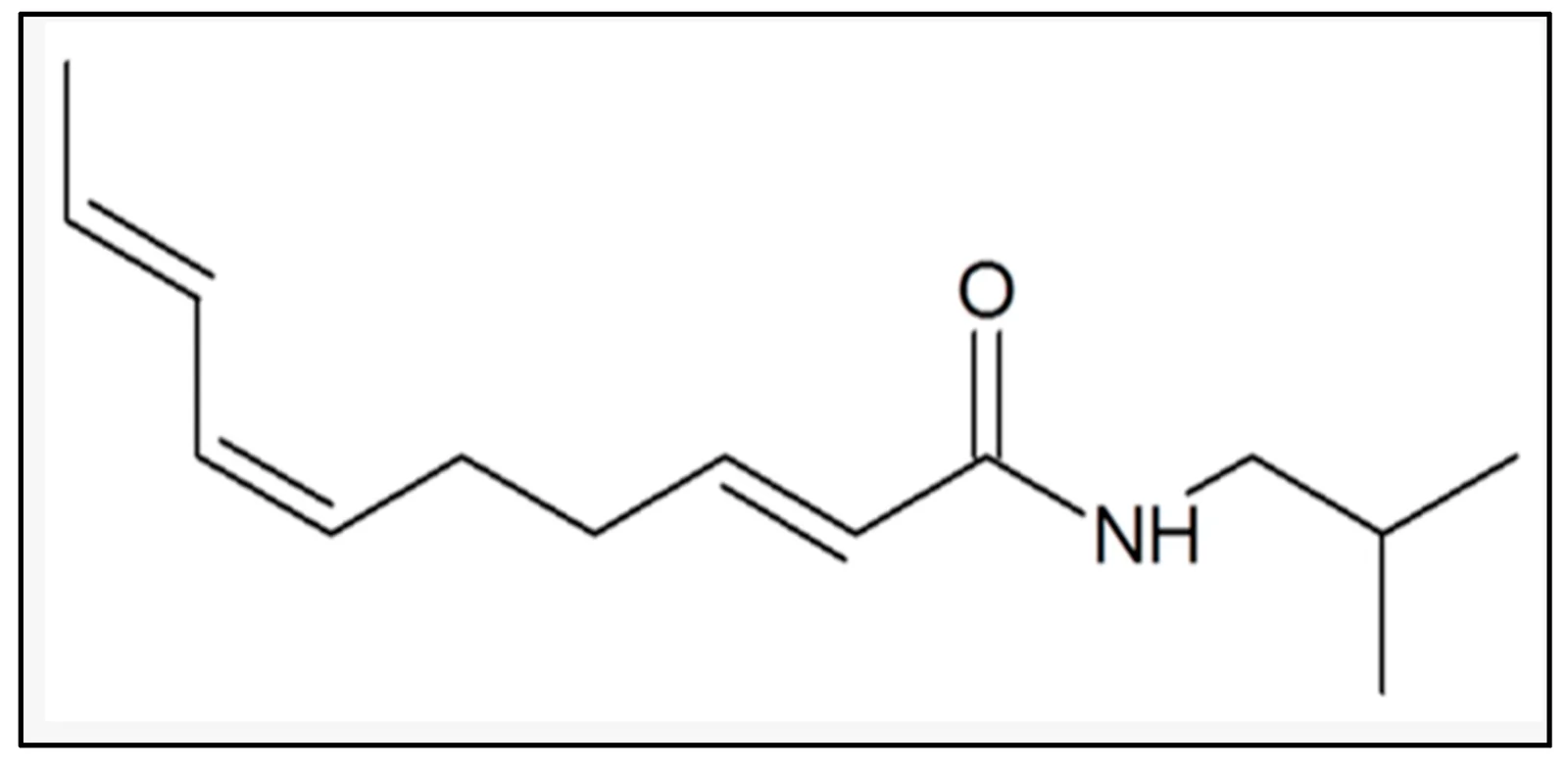
Representation of the alkamide affinin: structural formula of N-isobutyl affinin 2E,6Z,8E decatrien amide, the main bioactive component present in the crude lipid extract of the roots of chilcuague (*H. longipes*).

Adipose tissue is recognized as a dynamic endocrine organ that secretes adipokines and acts as a metabolic regulator. However, when it undergoes pathological overgrowth, it is directly linked to chronic diseases such as type 2 diabetes mellitus, cerebrovascular diseases, and certain types of cancer. Obesity, meanwhile, is caused by the excessive accumulation of triglycerides (TGs) in adipose tissue resulting from an imbalance between energy intake and expenditure [1,2]. Lipogenesis and adipogenesis are the primary processes involved in lipid production and accumulation within adipose tissue. These processes are promoted by various genes, transcription factors, and signaling molecules, such as fatty acid-binding protein (aP2), stearoyl-CoA desaturase-1 (SCD-1), CCAAT/enhancer-binding protein α (C/EBPα, δ, and β), peroxisome proliferator-activated receptor γ (PPARγ), and sterol regulatory element-binding protein-1c (SREBP-1c) [3]. Consequently, these signaling molecules, transcription factors, and enzymes represent important targets for the control and treatment of obesity. Therapies that regulate food intake remain the most effective clinical treatments for obesity, despite carrying the risk of severe side effects. Since reduced food intake is often accompanied by lower energy expenditure, the efficacy of these approaches and the likelihood of achieving sustained weight loss are limited. Understanding adipocyte differentiation and the therapeutic potential of adipose tissue as a pharmacological target positions it as a key organ or tissue for identifying drug targets within the adipocyte. Furthermore, traditional medicine has gained popularity in recent years, with herbal therapy usage increasing annually. Medicinal plants have been used for centuries as food and for medicinal purposes. Furthermore, various herbs possess anti-inflammatory and antioxidant properties and are used to treat disorders such as those caused by reactive oxygen species [3]. Medicinal plants and plant extracts represent the oldest and most widespread form of medicine. At least 25% of the active compounds in prescribed synthetic drugs were synthesized from active plant compounds [4,5]. Herbal and traditional medicine is emerging as an option for a growing number of people who purchase these products and explore their benefits [5,6]. A vast number of studies and trials have expanded our knowledge regarding the bioactive components of plant-based foods and their relationship with obesity. Polyphenols constitute one of the best-known groups of plant metabolites [7]. Studies have been conducted to investigate the anti-obesity effects of quercetin [6,8], epigallocatechin gallate (EGCG) [9], carotenoids such as fucoxanthin [10], and catechin-polyphenols, caffeine [11]. Likewise, metabolic stimulants—such as EGCG (a flavonoid) and Effects of red pepper on appetite and energy intake and capsaicin [12] have been studied, as have substances that increase energy expenditure and thermogenesis in humans. The use of natural products containing bioactive nutrients has been proposed as an effective solution to combat and even prevent obesity [12]. This could be a promising alternative strategy for developing safe and effective anti-obesity drugs in the future. A variety of natural products, including crude extracts and pure natural compounds, can induce weight loss and prevent diet-induced obesity. Various plant-derived products such as saponins, polyphenols, flavones, caffeine, and alkamides have been reported to possess anti-obesity activity [11,13,14]. Alkamides are bioactive compounds found in at least 33 plant families, formed by the linkage of an amine and a medium- or long-chain fatty acid (usually aliphatic) via an amide bond. Based on the type of unsaturated bonds, they are classified into olefinic alkylamides, containing at least one double bond, and acetylenic alkylamides, containing at least one triple bond. The significance of alkylamides lies in their status as bioactive compounds capable of triggering a notable response in receptor cells [15]. Another group of alkamides features homocyclic or heterocyclic rings at the terminal end of the chain; such structures are observed in capsaicin from certain chili peppers (*Capsicum* spp.) and piperine from black pepper (*Piper* spp.) [14,16,17]. The primary phytochemical in *H. longipes* roots is a bioactive alkylamide known as affinin [16,18,19], an unsaturated aliphatic isobutylamide (N-isobutyl-2E,6Z,8E decatrienamide) (Figure 1), also known as spilanthol. This substance was isolated in 1945 [20] and is present in species belonging to the genera *Heliopsis*, *Wedelia*, and *Spilanthes* within the Asteraceae family [15,20,21]. Biological effects such as local anesthetic activity, flavoring properties, and insecticidal and larvicidal activity [22,23], as well as antimicrobial, bactericidal, and fungistatic effects [23,24,25], have been characterized. Safety tests have evaluated acute toxicity, the median lethal dose (LD50 = 113 mg/kg), and the Ames test, revealing an absence of cytotoxic effects [26]. Furthermore, compounds such as capsaicin and other phytochemicals including alkylamides, trans-pellitorine and affinin exhibit anti-adipogenic activity both *in vitro* and *in vivo*, associated with the activation of the transient receptor potential vanilloid type 1 (TRPV1) [17,27,28,29,30]. Previous studies clearly indicate the potential local anti-obesogenic effect of affinin. The aim of the present study was to determine the effect of the alkamide affinin on key stages of adipocyte differentiation and glucose uptake in a 3T3-L1 adipocyte model of adipogenesis and insulin resistance.

## 2. Results

### 2.1. Determination of Non-Toxic Concentrations of Affinin Crude Extract in 3T3-L1 Cells

**Figure 2:**
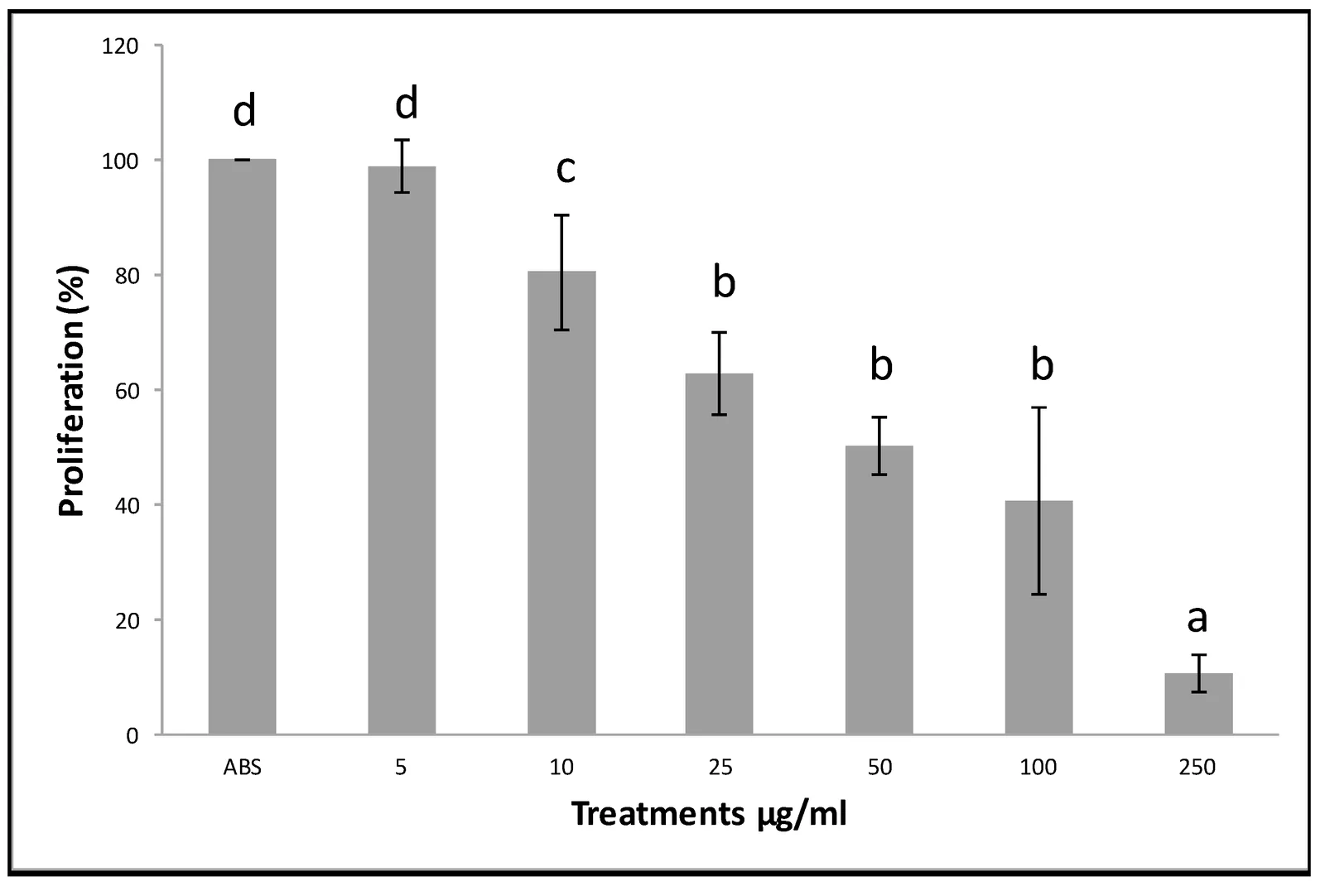
Effect of the crude extract of affinin on the proliferation of 3T3-L1 preadipocytes. 3T3-L1 preadipocytes were seeded in 24-well culture plates (1 × 10^4^ cells/well) in DMEM supplemented with 10% SFB (basal medium, BM). After 2 days, cultures were fed BM supplemented with different concentrations of affinin. After 24 h, the number of cells was determined by direct cell counting on a hemacytometer. The figures represent the mean value ± standard deviation (S.D) of four independent experiments. Lowercase letters indicate significant differences between treatments (Duncan, *p* ≤ 0.05).

To determine non-toxic concentrations of the crude affinin extract in 3T3-L1 preadipocytes, a concentration–response curve was generated. The highest concentrations evaluated—100, 150, and 250 µg/mL—reduced cell proliferation to less than 50%. Lower concentrations did not affect 3T3-L1 cell growth, as shown in Figure 2. The toxic concentration of the affinin extract was calculated using linear regression, with the equation y = −47.318x + 134.19 and a coefficient of determination of R^2^ = 0.9907. The IC50 value for the affinin extract was 60.150 µg/mL. Overall, these results indicate that the affinin extract can be cytotoxic at high concentrations. However, the maximum non-toxic concentration defined as the concentration resulting in less than 20% inhibition was established as 20 µg/mL.

### 2.2. Insulin-like Activity of Affinin Extract

**Figure 3:**
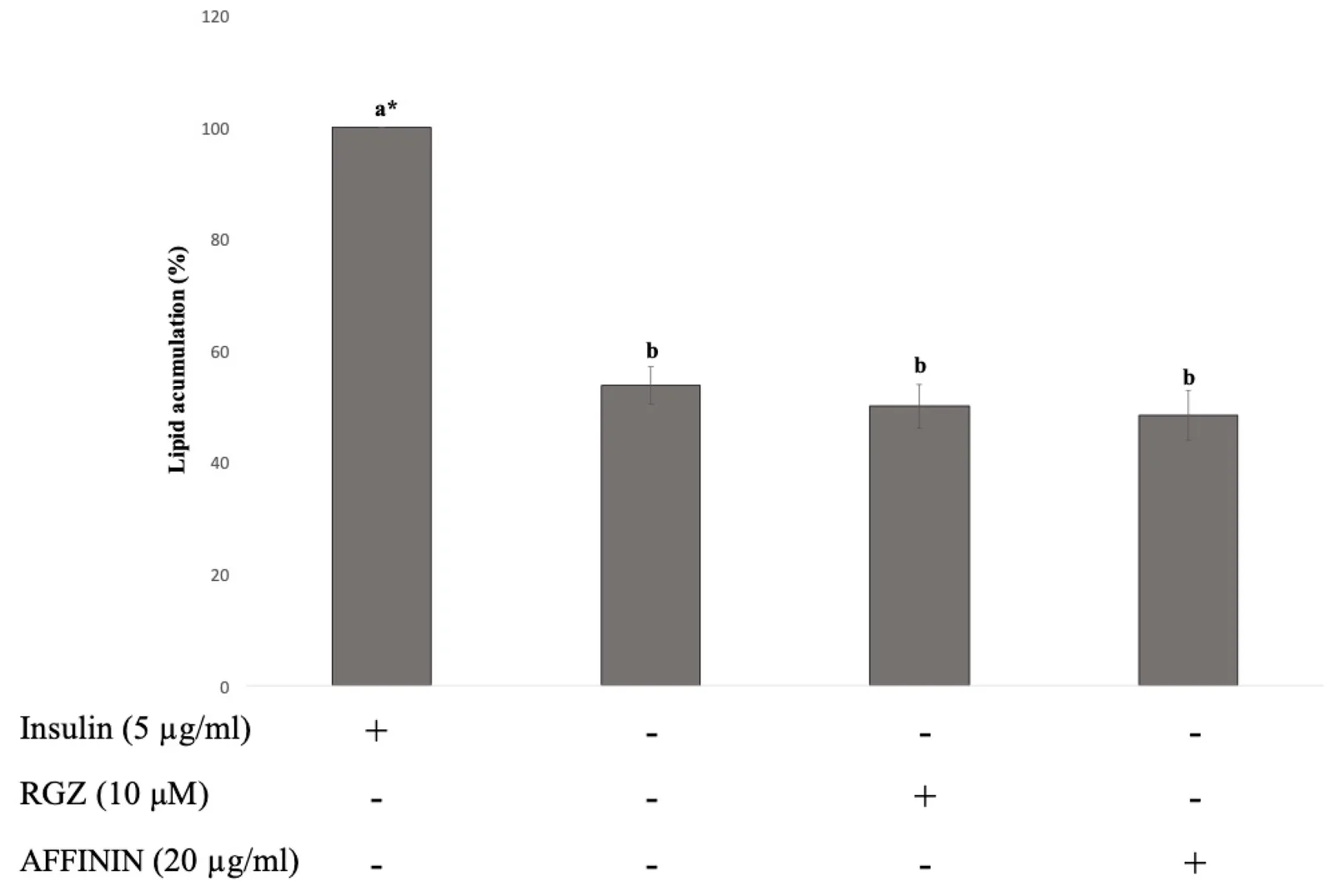
Effect of insulin-like activity of crude extract of affinin (20 μg/mL) on 3T3-L1 lipid accumulation. 3T3-L1 preadipocytes were seeded (3 × 10^5^ cells/well) in 35 mm plates in basal medium (BM) until preconfluence was achieved. Differentiation was induced with AM-I supplemented with 20 μg/mL of affinin extract, or RGZ (10 μM) or 5 μg/mL of insulin (100%); in 3T3-L1 cells, adipose conversion was estimated by quantitation of intracytoplasmic lipid accumulation. The results are presented as the mean ± SD of four independent experiments in quadruplicate. Lowercase letters indicate significant differences between treatments (Duncan, *p* ≤ 0.05). * Significant difference with respect to insulin-treated cells (Dunnett, *p* ≤ 0.05).

The effect of the affinin extract was evaluated in an adipogenic medium (AM) lacking insulin (AM-I^−^) to observe potential insulin-like activities that might stimulate adipogenesis. Under these conditions, the increase in triglycerides observed with the affinin extract was similar to that seen in untreated cells (negative control) (Figure 3) and in cells treated with the drug rosiglitazone (RGZ); the untreated control, RGZ, and the crude affinin extract all induced lipid accumulation amounting to 48% of that observed for insulin-treated control cells (100%). Undifferentiated preadipocytes were used as a negative control. This result suggests that the crude affinin extract does not induce adipogenesis in 3T3-L1 cells and does not exhibit insulin-like activity.

### 2.3. Effect of Affinin Extract on Triglyceride Accumulation in Insulin-Resistant and Sensitive 3T3-L1 Adipocytes

**Figure 4:**
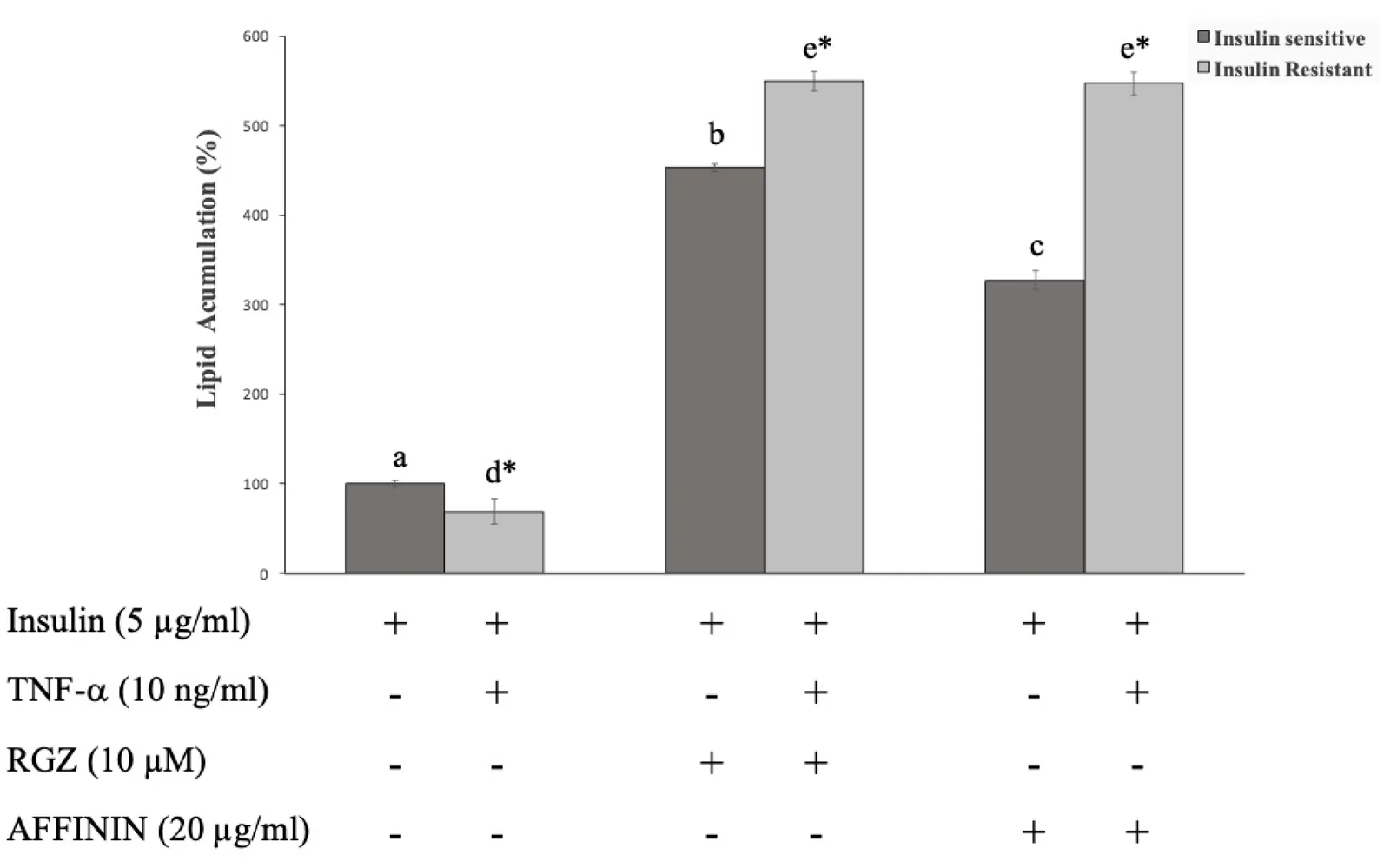
Effect of extracts of the alkamide affinin on the lipid accumulation of insulin-sensitive and insulin-resistant 3T3 adipocytes. Adipose differentiation was induced in preadipocytes with BM supplemented with 20 μg/mL of affinin extract. In parallel, another set of preadipocytes was treated with TNF-α 10 nM to induce insulin resistance. The results are represented as an average ± SD of at least four independent experiments in quadruplicate. Lowercase letters indicate significant differences between insulin-sensitive treatments (Duncan, *p* ≤ 0.05); (*) significant differences between insulin-sensitive and insulin-resistant cells of each treatment (t student, *p* ≤ 0.05).

To determine the effect of the crude affinin extract on insulin-sensitive and insulin-resistant adipocytes, the cytokine TNF-α (10 ng/mL) was used to induce insulin resistance. Subsequently, triglyceride accumulation in 3T3-L1 adipocytes was evaluated under various treatment conditions. Upon adding a non-toxic concentration of affinin extract (20 μg/mL) to the basal medium (BM), an increase in triglyceride accumulation was observed relative to the insulin-sensitive control. However, this increase was lower than that recorded in sensitive cells treated with rosiglitazone (RGZ, 10 μM), as shown in Figure 4. In insulin-resistant 3T3-L1 adipocytes, treatment with crude affinin extract (20 μg/mL) increased triglyceride content compared to the insulin-resistant control. Taken together, the results indicate that the crude affinin extract (20 μg/mL) enhances triglyceride accumulation in both insulin-sensitive and insulin-resistant adipocytes. Nevertheless, RGZ (10 μM) induced the greatest lipid accumulation under both experimental conditions.

### 2.4. Affinin Extract Stimulated the Uptake of 2-NBDG in Insulin-Sensitive and Insulin-Resistant Adipocytes

**Figure 5:**
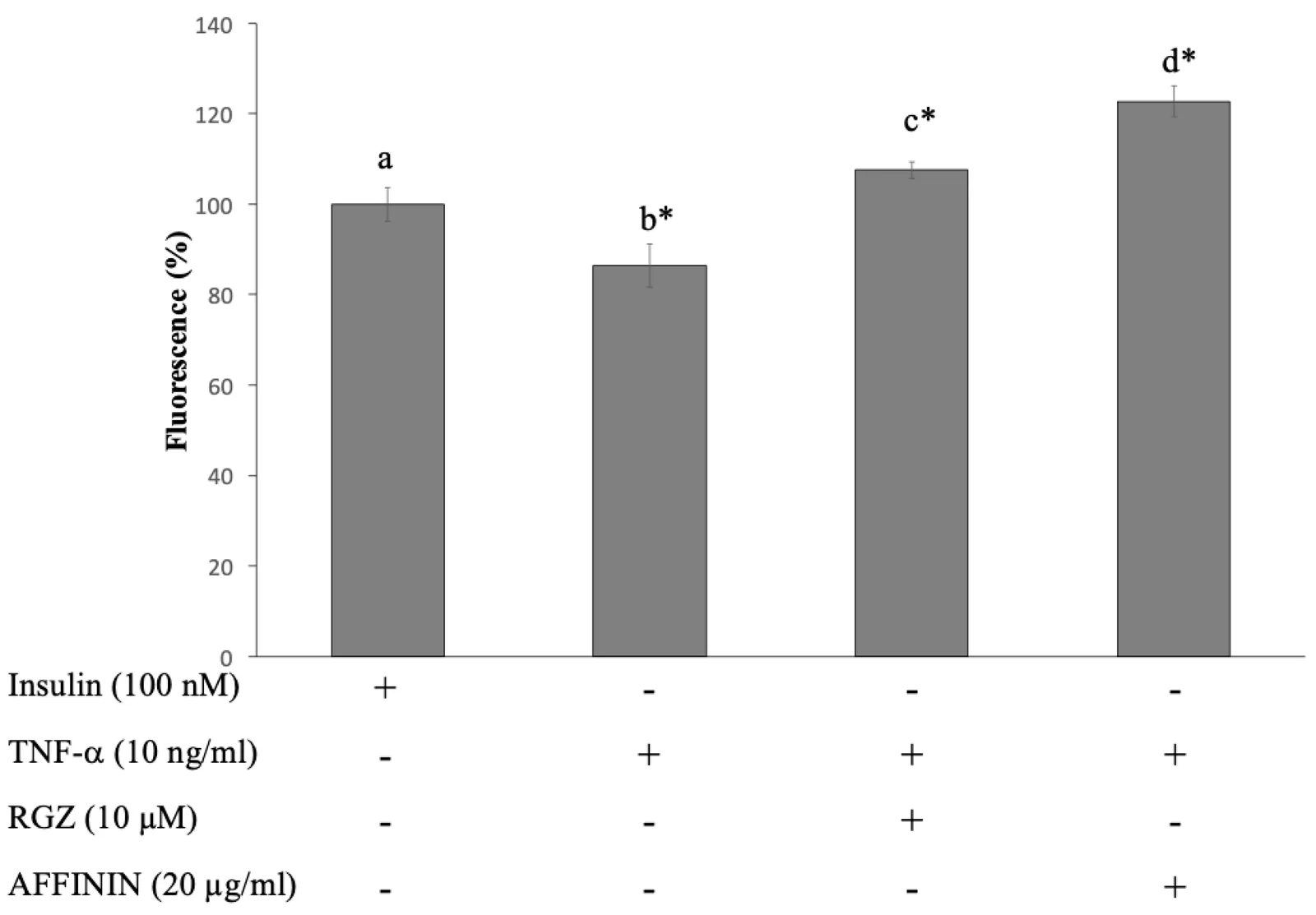
Effect of the alkamide affinin extract on 2-NBDG uptake in insulin-sensitive and insulin-resistant adipocytes. Mature 3T3-L1 adipocytes were treated with TNF-α to become insulin-resistant. The cells were incubated for 60 min with PBS/BSA containing 80 μM of 2-NBDG and 20 μg/mL of affinin. Control treatments received 100 nM insulin or rosiglitazone 10 μM. After incubation, free 2-NBDG was removed from the cultures and fluorescence associated with cell monolayers was measured. The results are presented as the average ± S.D. of three independent experiments in triplicate. Lowercase letters indicate significant differences between treatments (Duncan, *p* ≤ 0.05), (*) significant differences with respect to insulin-treated cells (Dunnett, *p* ≤ 0.05).

To determine whether the crude affinin extract stimulates glucose uptake in adipocytes, we evaluated its effect on the incorporation of 2-NBDG, a fluorescent glucose analogue, into differentiated 3T3-L1 adipocytes. The analysis was conducted using mature, differentiated, insulin-resistant adipocytes. Mature, differentiated, insulin-sensitive 3T3-L1 adipocytes, treated with 100 nM/mL insulin and not exposed to 10 ng/mL TNF-α, served as the control. In the assays, adipocytes treated with the crude affinin extract (20 μg/mL) incorporated 2-NBDG in response to insulin. Treatment with the affinin extract increased 2-NBDG uptake by 20% compared to the insulin-sensitive control cells. Rosiglitazone (RGZ, 10 μM) produced a moderate 10% increase relative to the insulin-sensitive control. The effect observed with RGZ was 10% lower than that recorded with the affinin extract in insulin-resistant adipocytes. These results suggest that the affinin extract promotes glucose uptake in insulin-resistant adipocytes and demonstrates an effect superior to that observed with the drug RGZ (Figure 5).

## 3. Discussion

Affinin, or spilanthol, is an alkamide-type secondary metabolite; in the plant *Heliopsis longipes*, these compounds are primarily located in the root. Various significant biological effects are attributed to these substances. *H. longipes* S. F. Blake (Asteraceae family) is a plant native to central Mexico that possesses medicinal properties; its roots are commonly known as chilcuague, pelitre, golden root, and Aztec root [31]. This species synthesizes various alkamides in its roots, with affinin being the most representative and abundant (~40% in organic extracts) [31,32]. This compound exhibits numerous biological activities [33], such as insecticidal and Molluscicidal [21], cytotoxic [26], larvicidal [23], antimicrobial and fungistatic [24,25], anti-inflammatory [33,34], analgesic [22], diuretic, and anti-adipogenic [35] properties. It modulates vasodilation [33,34,36,37,38]. It is a compound capable of permeating the oral mucosa [32], rapidly entering the bloodstream, and even crossing the blood–brain barrier [17,33]. From the perspectives of biology and the evaluation of traditional use, *Heliopsis longipes* is one of the most studied species in the genus. Furthermore, research groups have shown increasing interest in affinin supplementation, specifically regarding its application and effects on adipogenesis, the modulation of triglyceride synthesis, and glucose metabolism in both in vivo and in vitro models of obesity and type 2 diabetes mellitus (T2DM). Currently, the effects of various alkamides on adipogenesis and glucose uptake in preadipocytes and adipocytes are being investigated. Studies conducted in in vitro systems using preadipocytes treated with *Echinacea purpurea* flower extract, in which at least five distinct alkamides had previously been identified, showed an increase in PPAR-γ activity independent of adipocyte differentiation, as well as an increase in insulin-dependent glucose uptake. Separately, another study determined that the alkamide found in *Piper nigrum* reduced lipid accumulation during 3T3-L1 cell adipogenesis by blocking transient receptor potential (TRP) channels V1 and A1 [39,40]. Our study evaluated a crude extract of the alkamide affinin to identify its effects on typical adipogenesis parameters using the 3T3-L1 preadipocyte cell line; this line is well-characterized and considered a standard biological model for studying adipogenesis, insulin resistance, and type 2 diabetes mellitus. The crude extract of the alkamide affinin was provided by the Molecular Biology Laboratory of the UAQ Faculty of Natural Sciences (headed by Dr. Jesús Eduardo Castro Ruiz); the characterization, purification, and quantification procedure was previously reported by Castro Ruiz in 2017, and the extract used had a purity of 94% or higher [32]. First, the safety of the crude affinin extract was evaluated, and its maximum non-toxic concentration in 3T3-L1 preadipocytes was determined. To this end, concentration–response curves were generated, testing concentrations up to a maximum of 250 µg/mL. The toxic concentration, maximum non-toxic concentration, and working concentration were calculated via linear regression using the equation y = −47.318x + 134.19, with a coefficient of determination of R^2^ = 0.9907. Overall, the results indicate that the affinin extract may exhibit cytotoxicity at high concentrations. However, the concentration associated with less than 20% inhibition of proliferation was established as 20 µg/mL; therefore, this concentration was considered suitable for subsequent assays. Given that obesity is a side effect of some hypoglycemic agents [41], we first evaluated the effects of the affinin extract on 3T3-L1 cell adipogenesis. Our results show that the crude affinin extract lacks pro-adipogenic effects, as its addition to the differentiation medium (AM) did not alter triglyceride accumulation. Our results are consistent with previous reports indicating that affinin does not affect weight gain or visceral fat accumulation in mice [35,42]. Similarly, the results indicate that it does not induce adipogenesis in 3T3-L1 cells, a desirable property for hypoglycemic drugs. However, despite the absence of pro-adipogenic effects, the increased triglyceride accumulation observed in cells treated with the affinin extract—added to an insulin-free culture medium—suggests that the compound possesses insulin-like activity. We confirmed its insulin-mimetic properties by demonstrating 2-NBDG uptake in differentiated, insulin-resistant 3T3-L1 adipocytes. Affinin stimulated greater 2-NBDG uptake in 3T3-L1 adipocytes. Taken together, these results suggest that the affinin extract stimulated glucose uptake in mature, insulin-resistant adipocytes and lipid accumulation in preadipocytes—both effects characteristic of insulin action in the adipocyte lineage [28,29]. Given that 3T3-L1 fibroblasts are highly sensitive to insulin, they serve as a suitable model for studying adipogenesis and evaluating the effect of affinin on this process. To further explore the extract’s potential utility, we also analyzed its effects on 2-NBDG uptake in mature 3T3-L1 adipocytes treated with TNF-α to induce insulin resistance [43]. In these assays, TNF-α-treated 3T3-L1 adipocytes failed to take up 2-NBDG in response to insulin. However, they did actively take up this glucose analog following treatment with the affinin extract. Furthermore, the extract induced 20% greater 2-NBDG uptake than that observed in insulin-sensitive adipocytes and 10% greater uptake than that recorded with RGZ treatment (*p* ≤ 0.05). Among the possible mechanisms by which insulin-sensitizing agents related to adipogenesis and triglyceride synthesis exert their effects, the stimulation of glucose uptake by peripheral tissues represents one of the most promising therapeutic targets. Our results suggest that the affinin extract could exert anti-adipogenic and/or hypoglycemic effects by acting as an insulin-mimetic compound. This extract stimulated glucose uptake in insulin-resistant adipocytes without increasing lipid accumulation. Taken together, the ability of the affinin extract to promote glucose uptake in insulin-resistant adipocytes, combined with the absence of pro-adipogenic effects, indicates that this crude extract could be useful in processes related to the regulation of lipid and glucose metabolism. In a previous study, Huang et al. (2019) [35] evaluated the effects of affinin both in vivo and in vitro. That study observed that the compound did not affect weight gain or visceral fat accumulation in mice, nor did it promote adipogenic processes in affinin-treated 3T3-L1 preadipocytes. Furthermore, the authors reported that affinin exhibits anti-adipogenic properties and may contribute to ameliorating alterations associated with models of adipogenesis and insulin resistance. These findings support the possibility that affinin plays a role in regulating lipid and glucose metabolism. Similarly, studies conducted with alkamides from *Piper retrofractum* [44,45]. Kim and cols. showed a reduction in body weight, primarily attributed to a decrease in fat. Consistent with these findings, the results obtained in the present study show a reduction in triglyceride accumulation in 3T3-L1 adipocytes treated with the crude affinin extract from *H. longipes*. Taken together, these data suggest that the crude extract of affinin could attenuate alterations related to adipogenesis and insulin resistance through a mechanism of action like that described in previous studies [46,47].

## 4. Materials and Methods

The study was conducted in accordance with the Declaration of Helsinki, and all procedures and protocols of this research, as far as possible, were carried out following the approach and guidelines for non-animal models and methods used in the field of biomedical research; the replacement, reduction and refinement of animal testing (the “Three Rs”) of Directive 2010/63/EU on the protection of animals used for scientific purposes [48]. And the Good Cell Culture Practice Guidelines: A Report of the Second ECVAM Working Group on Good Cell Culture Practices [49].

### 4.1. Reagents, Cell Lines and Maintenance

The following chemicals were obtained from Sigma-Aldrich (St. Louis, MO, USA): Bovine Serum Albumin (BSA, Cat. No. A9418), insulin (CAS No: 11070-73-8, Cat. No. 1663), dexamethasone (CAS No: 50-02-2, Cat. No, D-2915), isobutyl-methyl-xanthine (CAS No: 28822-58-4, Cat. No: 15879) and rosiglitazone (RGZ) (CAS No: 122320-73-4: Cat. No: R-2408.) Dulbecco’s Eagle Modified Medium (DMEM, Thermo Fisher, Cat. No. 11965092); antibiotic–antimycotic solution (100, Thermo Fisher (Waltham, MA, USA), Cat. No. 15240096); Trypsin–EDTA solution (Thermo Fisher, Cat. No. R001100); and Fetal Bovine Serum (FBS, Thermo Fisher, Cat. No. 10082147) were all obtained from Gibco BRL (Grand Island, NY, USA). The glucose analogue 2-[N-(7-Nitrobenz-2oxa-1,3-diazole-4-yl) amino]-2-deoxy-D-glucose (2-NBDG) was acquired from Invitrogen (Carlsbad, CA, USA). Tumor Necrosis Factor-Alpha (TNF-α) was acquired from Prepotech (London, UK). Triglycerides were quantified by the colorimetric method (BioViosion Inc., Hercules, CA, USA). 3T3-L1 fibroblasts were acquired from ATCC, ATCC CL-173^TM^ (Manassas, VA, USA). Freezing, cultures, subcultures, ducts, and cryopreservation methods were performed following the instructions recommended by the provider in the ATCC protocols for this cell line, protocols 12.1 [50], 12.2 [51], and 12.3 [52], and Fresnhey’s technical manual [52,53]. The experiments were conducted in subcultures of passages 4–10. The cultures were kept at 37 °C in a humidified atmosphere of 5% CO_2_ with changes of medium every two days.

### 4.2. Plant Material

The crude extract of the alkamide affinin (purity > 94%) was provided by the Cell and Molecular Biology Laboratory of the Faculty of Natural Sciences at the Autonomous University of Querétaro, where it had been characterized and purified following protocols described by Castro-Ruíz in 2017 [32]. A dichloromethane extract was used to isolate the bioactive compounds, as it offered a higher yield (19 g/kg dry root weight) compared to the ethanolic extract (17 g/kg dry root weight). This extract (100 g) was fractionated via flash open-column chromatography to obtain 21 fractions. Subsequent chromatography resulted in the isolation of 28.5 g of pure affinin. Affinin was identified using proton and carbon-13 nuclear magnetic resonance spectroscopy (1H-NMR and 13C-NMR) [20]. Purity analysis via reversed-phase high-performance liquid chromatography with a photodiode array detector (HPLC-PDA) revealed a purity of >94% [32]. The lyophilized extract was stored under refrigeration, protected from light, until use in cell cultures. Solutions were prepared in dimethyl sulfoxide (DMSO) and sterilized by filtration using 0.22 μm filters (Millipore^®^, Billerica, MA, USA). Subsequently, they were diluted in culture medium under sterile conditions. Dilutions were freshly prepared in DMEM supplemented with 10% fetal bovine serum and 1% antibiotic–antimycotic solution. In all cases, the final concentration of the solvent (DMSO) did not exceed 0.5%. The final stock solution was prepared at a maximum concentration of 250 μg/mL.

### 4.3. Determination of Non-Toxic Concentrations for Affinin Crude Extract

3T3-L1 preadipocytes were seeded (1 × 10^4^ cells/well) in 24-well plates (Corning Glass Works, Corning, NY, USA) with DMEM supplemented with 10% FBS (basal medium, BM). Cell cultures were kept at 37 °C in an atmosphere humidified at 5% CO_2_. Two days later, cultures were fed with BM supplemented with different concentrations of affinin crude extract and cell viability was evaluated at 24 h by direct cell counting in a hemacytometer.

### 4.4. Effect of the Alkamide Affinin Crude Extract on Adipogenesis

3T3-L1 preadipocytes were seeded (3 × 10^5^ cells/well) in 35 mm plates in BM until preconfluence was reached and then treated with adipogenic medium (AM; DMEM containing 10% FBS, 5 μg/mL insulin, 0.5 μM IBMX, 0.4 μg/mL dexamethasone) [43,54,55] supplemented with non-toxic concentrations of the crude extract of affinin (20 μg/mL) or without treatment as a negative control. Positive control cells were treated with 5 μg/mL insulin or 5 μg/mL insulin supplemented with the hypoglycemiant RGZ 10 μM. After 7 days, the triglyceride accumulation was estimated by spectrometry following the manufacturer’s instructions (Triglyceride Quantification Kit Catalog #K622-100, BioVision Inc., Milpitas, CA, USA).

### 4.5. Determination of Insulin-like Activity of Affinin Crude Extract

Preconfluent 3T3-L1 preadipocytes with 70 to 80% confluence were treated with adipogenic medium lacking insulin (AM-I) supplemented with non-toxic concentrations of the crude extract of affinin. Positive control cultures were treated with 5 µg/mL insulin or 10 µM RGZ, whereas negative control cells did not receive this treatment. After 7 days, the insulin-like activity was estimated as the degree of lipid accumulation by spectrometry, as described following the manufacturer’s instructions (Triglyceride Quantification Kit Catalog #K622-100, BioVision Inc.).

### 4.6. Effect of Affinin Crude Extract on 2-NBDG Uptake in 3T3-L1 Adipocytes

Differentiated 3T3-L1 adipocytes, cultured on 96-well fluorescence plates, were incubated for 60 min with PBS containing 1 mg/mL of BSA and 80 μM of the fluorescent glucose analog 2-NBDG [56,57] in the presence of non-toxic concentrations of the crude extract of affinin. Control cultures were treated with insulin at 100 nM or with 10 μM of RGZ. In parallel experiments, 3T3-L1 adipocytes were previously incubated for 4 additional days with TNF-α 10 ng/mL to induce insulin resistance [54] and then used for 2-NBDG uptake experiments. After incubation, cultures were washed out and fluorescence retained in cell monolayers was measured with a fluorescence microplate reader (Thermo-Scientific Varioskan Flash, Waltham, MA, USA) with excitation and emission wavelengths of 485 nm and 535 nm, respectively, using the software SkanIt 5.2.4.5. Values of 2-NBDG incorporation in the absence of insulin were subtracted from those obtained with insulin at 100 nM to establish 100% specific 2-NBDG incorporation.

### 4.7. Effect of Affinin Crude Extract on Lipid Synthesis in Both Insulin-Sensitive and Insulin-Resistant 3T3-L1 Adipocytes

Preconfluent 3T3-L1 preadipocytes were treated with AM supplemented with non-toxic concentrations of the crude extract of affinin (20 μg/mL) or without treatment as negative control. In parallel experiments, 3T3-L1 adipocytes were simultaneously incubated with AM supplemented with TNF-α at 10 ng/mL to induce insulin resistance [54,58], and non-toxic concentrations of the crude extract of affinin, or without treatment as a negative control. In both cases, the positive control cultures were treated with RGZ at 10 μM. After 7 days, the intracellular triglyceride accumulation was estimated by spectrometry following the manufacturer’s instructions (Triglyceride Quantification Kit Catalog #K622-100, BioVision Inc.).

### 4.8. Data Analysis

The data from the studies were collected and graphed using the Microsoft Excel^®^ 2007 (version 12.0) software (Microsoft Corporation, Redmond, WA, USA). Cell proliferation values were calculated as percentage values relative to controls (defined as 100%) and are expressed as the mean ± standard deviation. A logarithmic scale was used for the range of concentrations used, and linear regression was calculated according to protocols 24.1 and 24.4 from Freshney. The IC_50_ and IC_20_ values were obtained by linear regression analysis. Toxicity tests were performed in quadruplicate, and the figures represent the mean ± SD value of four independently conducted experiments. To carry out a statistical analysis that would allow for detecting significant differences between the treated samples and the control samples, at least four independent experiments were carried out, varying the final total number depending on the parameter evaluated and the type of methodology used for their study. Data were analyzed by using one-way ANOVA. The level of *p* ≤ 0.05 was used as a criterion of statistical significance between treatments (Duncan) or between each treatment with respect to the control (Dunnett). All calculations were performed using SPSS (Statistical Package for the Social Sciences) version 15.1 (SPSS, Chicago, IL, USA).

## 5. Conclusions

This report presents scientific evidence regarding the anti-adipogenic effect of a crude affinin extract, contributing to an understanding of its impact on adipogenesis and glucose uptake in highly insulin-sensitive cells (adipocytes), as well as in a cellular model of insulin resistance. Experimental evidence suggests that this effect is primarily driven by affinin, making it a promising molecule for treating cardiovascular conditions such as obesity and type 2 diabetes. However, further studies are needed to better understand the molecular pathways and the mechanism of action.

## Data Availability

The original contributions presented in this study are included in the article. Further inquiries can be directed to the corresponding author.

## Abbreviations

The following abbreviations are used in this manuscript:

2-NBDG	2-[N-(7-Nitrobenz-2oxa-1,3-diazole-4-yl)amino]-2-deoxy-D-glucose
DMEM	Dulbecco’s Eagle Modified Medium
FBS	Fetal Bovine Serum
BSA	Bovine Serum Albumin
AM	Adipogenic Medium
AM-I	Insulin-free Adipogenic Medium
